# Low-Density Lipoprotein Cholesterol Target Attainment in Patients With Established Cardiovascular Disease: Analysis of Routine Care Data

**DOI:** 10.2196/16400

**Published:** 2020-04-02

**Authors:** T Katrien J Groenhof, Daniel Kofink, Michiel L Bots, Hendrik M Nathoe, Imo E Hoefer, Wouter W Van Solinge, A Titia Lely, Folkert W Asselbergs, Saskia Haitjema

**Affiliations:** 1 Julius Center for Health Sciences and Primary Care University Medical Center Utrecht Utrecht University Utrecht Netherlands; 2 Division of Heart and Lungs, Department of Cardiology University Medical Center Utrecht Utrecht University Utrecht Netherlands; 3 Laboratory of Clinical Chemistry and Hematology University Medical Center Utrecht Utrecht University Utrecht Netherlands; 4 Department of Obstetrics, Wilhelmina Children’s Hospital University Medical Center Utrecht Utrecht University Utrecht Netherlands; 5 Institute of Cardiovascular Science Faculty of Population Health Sciences University College London London United Kingdom; 6 Health Data Research UK Institute of Health Informatics University College London London United Kingdom

**Keywords:** learning health care system, routine clinical data, cardiovascular risk management, LDL-c

## Abstract

**Background:**

Direct feedback on quality of care is one of the key features of a learning health care system (LHS), enabling health care professionals to improve upon the routine clinical care of their patients during practice.

**Objective:**

This study aimed to evaluate the potential of routine care data extracted from electronic health records (EHRs) in order to obtain reliable information on low-density lipoprotein cholesterol (LDL-c) management in cardiovascular disease (CVD) patients referred to a tertiary care center.

**Methods:**

We extracted all LDL-c measurements from the EHRs of patients with a history of CVD referred to the University Medical Center Utrecht. We assessed LDL-c target attainment at the time of referral and per year. In patients with multiple measurements, we analyzed LDL-c trajectories, truncated at 6 follow-up measurements. Lastly, we performed a logistic regression analysis to investigate factors associated with improvement of LDL-c at the next measurement.

**Results:**

Between February 2003 and December 2017, 250,749 LDL-c measurements were taken from 95,795 patients, of whom 23,932 had a history of CVD. At the time of referral, 51% of patients had not reached their LDL-c target. A large proportion of patients (55%) had no follow-up LDL-c measurements. Most of the patients with repeated measurements showed no change in LDL-c levels over time: the transition probability to remain in the same category was up to 0.84. Sequence clustering analysis showed more women (odds ratio 1.18, 95% CI 1.07-1.10) in the cluster with both most measurements off target and the most LDL-c measurements furthest from the target. Timing of drug prescription was difficult to determine from our data, limiting the interpretation of results regarding medication management.

**Conclusions:**

Routine care data can be used to provide feedback on quality of care, such as LDL-c target attainment. These routine care data show high off-target prevalence and little change in LDL-c over time. Registrations of diagnosis; follow-up trajectory, including primary and secondary care; and medication use need to be improved in order to enhance usability of the EHR system for adequate feedback.

## Introduction

At present, quality of care is generally evaluated in clinical trials or in expensive and laborious cross-sectional studies, such as the European Action on Secondary and Primary Prevention by Intervention to Reduce Events (EUROASPIRE) or SUrvey of Risk Factor management (SURF) initiatives, which evaluated target attainment of low-density lipoprotein cholesterol (LDL-c) [1-3]. These studies estimated the proportion of LDL-c target attainment and showed the magnitude of the clinical problem on a patient population level but did not provide feedback on an individual patient level. Also, generalizability may be limited due to selection bias of the studied population and/or selective nonresponse of patients. This has sparked interest in the use of routine care data for research purposes [4]. Routine care data better reflects the real-world situation and is less affected by nonresponse. This improves generalizability of results and makes routine care data more suitable for prevalence questions as compared to clinical trial or dedicated cohort data [4]. Moreover, it provides a continuously updated dataflow of a large amount of clinically relevant information at low costs. Finally, it allows direct feedback to treating physicians on performance and potentially allows benchmarking within a similar group of physicians. This is part of the development of a learning health care system (LHS) [5], in which routine clinical care and science are aligned via a constant cycle of data assembly, data analysis, interpretation, feedback, and change implementation [6].

Cardiovascular risk management (CVRM) is an example for complex care, with many factors and physicians involved over a long period, that could benefit from an LHS approach. Risk-factor level reduction and control is key in primary and secondary cardiovascular risk prevention. In particular, pharmacological LDL-c-lowering treatment is one of the cornerstones of cardiovascular disease (CVD) prevention, leading to a large risk reduction [7]. However, LDL-c management is far from optimal, as many patients fail to reach their appropriate LDL-c target values [8]. In cross-sectional analyses in patients on statin treatment, more than 80% of patients did not reach their LDL-c targets [1]. However, information on trends over time and factors associated with improvement or deterioration of LDL-c are lacking.

In this study, we evaluated the potential of routine clinical care data extracted from electronic health records (EHRs) to obtain reliable information on LDL-c management in CVD patients referred to a tertiary care center.

## Methods

### Study Design

We conducted a prospective study with data extracted from the EHRs of patients of the University Medical Center (UMC) Utrecht, Utrecht, the Netherlands. All data from the EHRs of the UMC Utrecht are stored in the Utrecht Patient-Oriented Database (UPOD). In short, this database comprises all clinical information, demographic data, medication, diagnoses, and lab measurements, directly extracted from the EHRs of patients who visited the UMC Utrecht from 2003 onward, encompassing data from more than 2 million individual patients to date [9]. A complete description of the UPOD database has been published elsewhere [9]. The use of EHR data is in accordance with Institutional Review Board and privacy regulations of the UMC Utrecht: clinical data can be used for scientific purposes if patients cannot be identified directly from the data. All patients were informed on the opt-out procedure, a general UMC Utrecht procedure through which patients can object to use of their clinical data for scientific evaluations. A waiver was obtained for this study from the Institutional Review Board. We used data collected from February 2003 to December 2017.

### Study Population

All patients with at least one documented LDL-c measurement in the database were included in the study. This study’s analysis was restricted to patients with established CVD, as these patients have an indication for LDL-c management according to the Dutch guidelines [10]. Established CVD was defined as a history of coronary heart disease, stroke, peripheral artery disease, or abdominal aortic aneurysm based on diagnosis codes; interventions, including operative procedures and stenting; and financial billing codes (available upon request). We applied a window of 1 week before and 1 week after the date of the LDL-c measurement for the CVD status to include measurements that were part of a preoperative screening. Quality check of the CVD detection algorithm in a subset of patients (n=20) showed 100% accuracy for labelling an individual as a patient with established CVD.

### Data Extraction and Appraisal

All LDL-c measurements in adult patients (≥18 years of age) available at the UMC Utrecht were retrieved from the UPOD. In patients for whom all other lipids but LDL-c were measured, LDL-c was calculated using the Friedewald formula [11]. Before January 24, 2017, the UMC Utrecht laboratory only used the Friedewald formula to calculate LDL-c. Since LDL-c values below 0.8 mmol/L and/or triglyceride values over 8.0 mmol/L are considered unreliable when using the Friedewald formula, these values were considered unreliable and were therefore excluded. From January 24, 2017, onward, the laboratory started manual remeasurement of LDL-c values below 0.8 mmol/L. Therefore, LDL-c values after January 24, 2017, that were below 0.8 mmol/L were included in this analysis.

We extracted information on sex, age, diabetes mellitus, hypertension, chronic kidney disease (CKD), blood pressure, smoking status, and use of blood pressure-lowering, lipid-lowering, or blood glucose-lowering medication. Sex and age were extracted from the general hospital administration data, which are checked via identification during the first visit at our center. History of diabetes mellitus was based on diagnosis codes, financial billing codes, and prescription of blood glucose-lowering medication. Hypertension was defined as blood pressure over 140/90 mmHg and/or prescription of blood pressure-lowering medication. CKD was defined using diagnose codes; interventions, including dialysis and shunt surgery; or estimated glomerular filtration rate levels that were extracted from the laboratory system within 48 hours around the LDL-c measurement. Smoking status was retrieved from predefined tables, dedicated to smoking registration, as well as from free text. Blood pressure-lowering, lipid-lowering, blood glucose-lowering, and antithrombotic medication data were extracted from the electronic prescription system using the Anatomical Therapeutic Chemical classification codes starting with A10, B01, B02A, and C02-C10. We converted statin dosages to atorvastatin 20 mg equivalent dosages (see Table MA1-1 in Multimedia Appendix 1) to be able to assess differences in statin doses.

### Patient Selection

After extracting LDL-c measurements from the database, we excluded patients with unreliable LDL-c values, as described above, and patients without established CVD. We divided the remaining group into patients with repeated measurements and patients without repeated measurements.

### Data Analyses

First, we calculated the prevalence of target attainment at the first measurement per patient, which was the only measurement for the patients without repeated measurements. The LDL-c target was defined as less than 2.5 mmol/L, according to the Dutch CVRM guideline [10]. Also, we calculated the prevalence of LDL-c measurements within the following categories: on target or less than 0.5 mmol/L, 0.5-0.9 mmol/L, 1.0-1.4 mmol/L, 1.5-1.9 mmol/L, or more than 2.0 mmol/L off target. These distributions were compared between patients with and without repeated measurements. Additionally, we performed a logistic regression analysis to assess associations of elevated LDL-c levels at the first measurement with age, sex, diabetes, hypertension, CKD, statin use, antithrombotic agent use, smoking, and having repeated measurements (*yes* or *no*).

**Figure 1:**
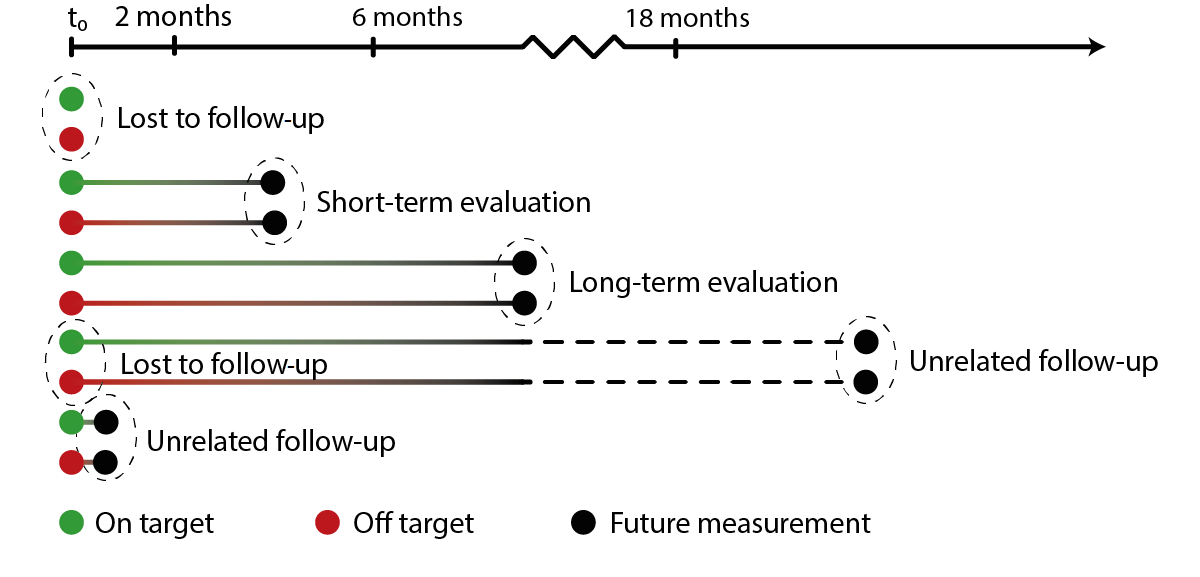
Visualization of possible follow-up scenarios.

Second, we investigated the trajectories of LDL-c distributions in patients with repetitive measurements. For the repetitive measurements, we distinguished different follow-up scenarios (see Figure 1) as follows: short-term evaluation (within 2-6 months from the previous measurement), long-term evaluation (within 6-18 months from the previous measurement), and unrelated follow-up. Unrelated follow-up measurements were measurements that followed either too short or too long after the previous measurement to be related to that measurement in terms of clinical evaluation; according to the guidelines, new therapy has to be evaluated after 3 months and yearly if medication remains the same [10]. These unrelated measurements were excluded from the trajectory analyses. Using the TraMineR package from R statistical software, version 4.3 (The R Foundation), we extracted trajectories, or *state sequences*, of the patients. A state sequence is defined as the order of different states, with states being one of the LDL-c categories (on target or <0.5 mmol/L, 0.5-0.9 mmol/L, 1.0-1.4 mmol/L, 1.5-1.9 mmol/L, or >2.0 mmol/L off target). Transition probabilities were calculated for LDL-c categories between measurement pairs. The first measurement can be the first of the sequence as a whole, where we then calculate the probability to transit into a certain LDL-c category at the second measurement; however, the first measurement can also be the second measurement of a sequence, where the transition probability to a category at the third measurement is calculated. To analyze clustering among state sequences, we made a subselection truncated at the 75th percentile of the total number of measurements per individual (ie, 6 or less measurements). Dissimilarity was calculated via optimal matching between sequences, and similar sequences were regrouped using cluster analysis. Per cluster, associations with covariates were analyzed using a generalized linear model with the clusters as the outcome and covariates of interest as the explanatory variables.

Lastly, we assessed factors associated with unfavorable LDL-c category change. Favorable change was defined as an LDL-c decreasing to or remaining on target. Unfavorable change was defined as an increase in LDL-c, a decrease in LDL-c but still off target, or a stable LDL-c that was off target. We performed a logistic regression analysis with deterioration as the outcome and age, sex, diabetes, hypertension, smoking, antithrombotic agent use, statin change (type and dose), the number of the measurement, and follow-up time (short- or long-term) as covariates.

All analyses were performed in R statistical software, version 4.3 (The R Foundation).

## Results

### Patient Selection

**Figure 2:**
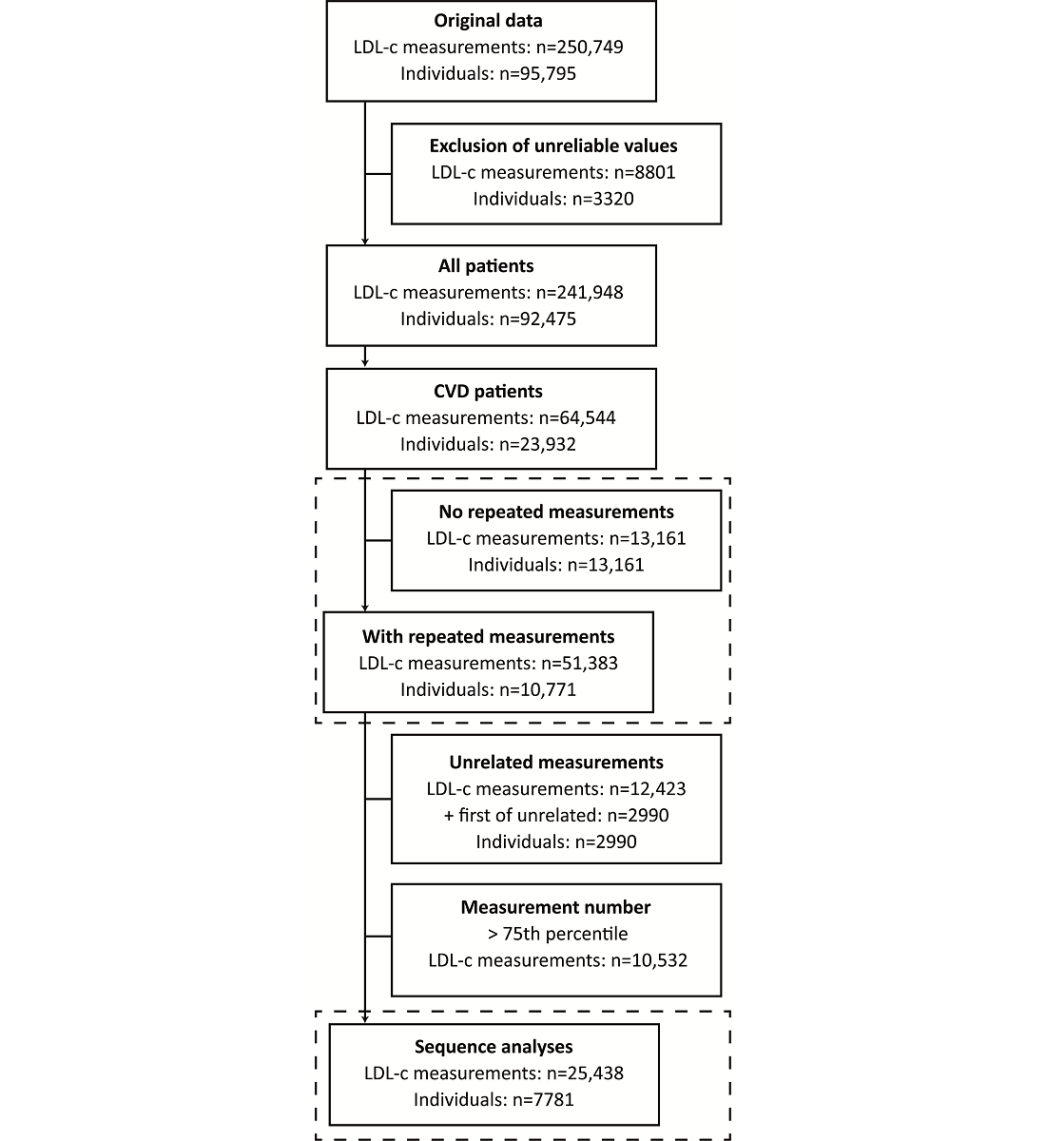
Flowchart of data retrieval for the study. CVD: cardiovascular disease; LDL-c: low-density lipoprotein cholesterol.

A total of 250,749 LDL-c measurements were collected from 95,795 individual patients at the UMC Utrecht between February 2003 and December 2017 (see Figure 2). We excluded 8801 LDL-c measurements from 3320 patients because of unreliable values (LDL-c <0.8 mmol/L and/or triglycerides >8.0 mmol/L). This left us with 241,948 LDL-c measurements from 92,475 individual patients. Of these, 23,932 patients (25.88%) had established CVD at the time of the LDL-c measurement.

### First Low-Density Lipoprotein Cholesterol Measurements

In 23,932 patients with CVD, LDL-c was measured repeatedly in 10,771 patients (45.00%) and once in 13,161 patients (54.99%) (see Table 1). The prevalence of target attainment was, on average, 48%: target attainment occurred in 4632 of 10,771 (43.00%) patients with repeated measurements and in 6844 of 13,161 (52.00%) patients without repeated measurements, which was stable over the years from 2003 to 2017 (see Table MA1-2 in Multimedia Appendix 1).

**Figure 3:**
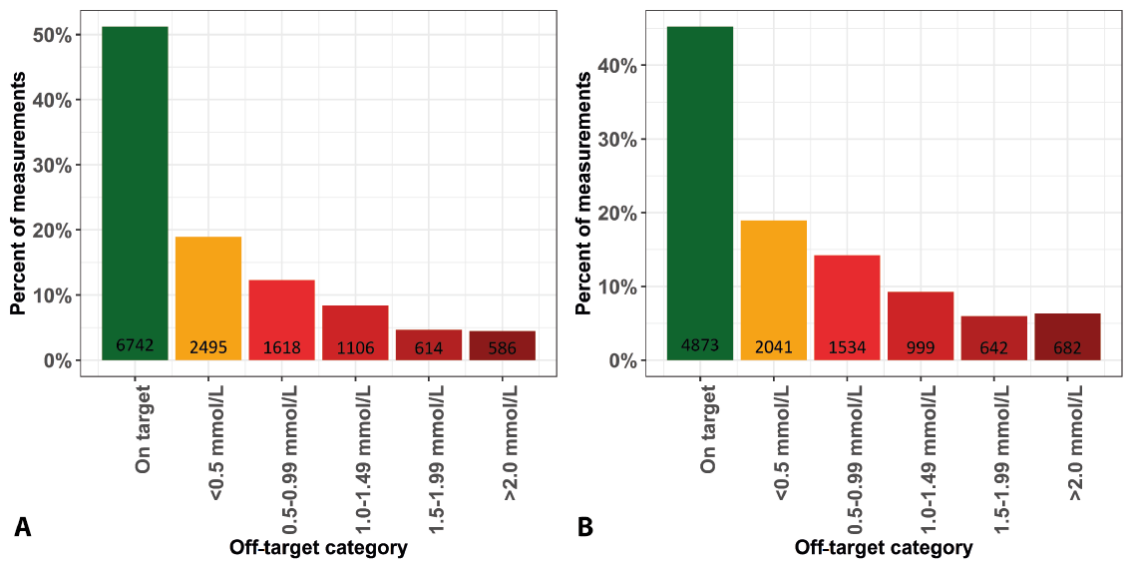
Low-density lipoprotein cholesterol distributions stratified for patients with and without repeated measurements. A. Patients without repeated measurements. B. Patients with repeated measurements. Values on the x-axes represent mmol/L from the target.

The distributions of LDL-c categories (see Figure 3 A and B) were similar for patients with and without repeated measurements. Patients with repeated measurements were younger (mean 60.8 years, SD 12.1, vs mean 65.5 years, SD 12.8, *P*<.001). Cardiovascular medication use—lipid lowering, blood pressure lowering, blood glucose lowering, or antithrombotic—was, on average, extracted from 51% of patients.

**Table 1:** Baseline characteristics for cardiovascular disease (CVD) patients at first measurement in strata of presence of repeated measurements.

Characteristic		No repeated measurements (N=13,161)	Repeated measurements (N=10,771)
Women, n (%)		4257 (32.35)	3254 (30.21)
Age (years), mean (SD)		65.5 (12.8)	60.8 (12.1)
Smoking (current), n (%)		1523 (11.57)	967 (8.98)
LDL-c^a^ (mmol/L), median (IQR)		2.4 (1.9-3.1)	2.4 (1.9-3.1)
Systolic blood pressure (mmHg), mean (SD)		137.5 (23.5)	135.3 (23.2)
Diastolic blood pressure (mmHg), mean (SD)		76.3 (13.5)	77.5 (13.7)
Diabetes, n (%)		1456 (11.06)	1415 (13.14)
Hypertension, n (%)		4428 (33.64)	3514 (32.62)
Chronic kidney disease, n (%)		43 (0.33)	108 (1.00)
**Prevalent CVD, n (%)**			
	Coronary heart disease	9313 (70.76)	7660 (71.11)
	Stroke	2912 (22.13)	1929 (17.91)
	Peripheral artery disease	1461 (11.10)	1791 (16.63)
	Abdominal aortic aneurysm	502 (3.81)	503 (4.67)
**Registered medication, n (%)**			
	Statin	4616 (35.07)	3368 (31.27)
	Other lipid lowering	59 (0.45)	32 (0.30)
	Blood pressure lowering	5690 (43.23)	4193 (38.93)
	Glucose lowering	1065 (8.09)	685 (6.36)
	Antithrombotic	5863 (44.55)	4329 (40.19)

^a^LDL-c: low-density lipoprotein cholesterol.

In multivariable logistic regression analysis, more women were off target (odds ratio [OR] 1.48, 95% CI 1.40-1.56) compared to men (see Table 2). Patients with a history of hypertension or diabetes were more often on target (OR 0.87, 95% CI 0.83-0.92, and OR 0.69, 95% CI 0.55-0.65, respectively), as were statin users (OR 0.86, 95% CI 0.80-0.93). Smokers and patients with repeated measurements were more likely to be off target. No difference was found for patients with CKD nor for patients using antithrombotic medications.

**Table 2:** Logistic regression: factors associated with being off target at first measurement.

Characteristic		Odds ratio (95% CI)^a^
Age (per-year increase)		0.99 (0.98-0.99)
Women		1.48 (1.40-1.56)
Diabetes		0.69 (0.55-0.65)
Hypertension		0.87 (0.83-0.92)
Chronic kidney disease		0.75 (0.54-1.04)
**Medication**		
	Statin use	0.86 (0.80-0.93)
	Antithrombotic	0.98 (0.91-1.05)
Smoking		1.29 (1.19-1.41)
Repeated measurements		1.25 (1.19-1.32)

^a^Total number of patients was 23,932.

### Trajectory Analyses

**Figure 4:**
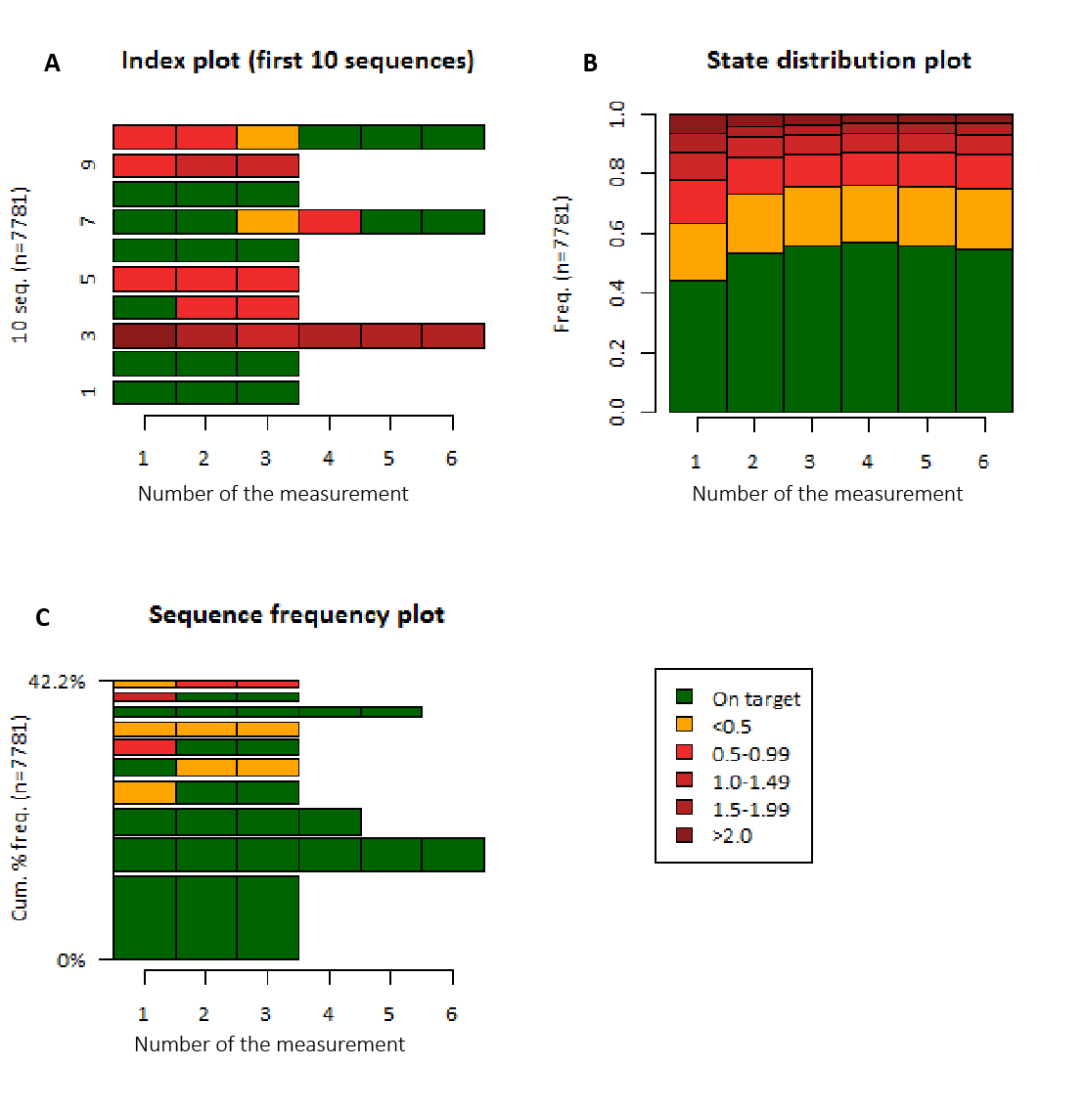
State sequences of low-density lipoprotein cholesterol (LDL-c) categories. A. Example of the sequences from the first 10 patients in the dataset (10 seq.). B. State distributions (equal to prevalence of LDL-c categories) per measurement. C. Most common sequences. LDL-c values in the legend are in mmol/L. Cum % freq: cumulative percentage frequency; Freq: frequency.

We extracted 51,383 repetitive measurements from 10,771 patients. Of these, 12,423 measurements (24.18%) were unrelated and, thus, excluded, leaving only one measurement for 2990 patients, which were also excluded. The number of measurements ranged from 2 to 40. After truncation of the measurements at the 75th percentile (number of measurements was 6), 25,438 LDL-c measurements in 7781 patients remained for the cluster analysis. State sequences, of which an example of 10 is shown in panel A from Figure 4, were calculated. State distributions (ie, the distribution of LDL-c categories per measurement number) are shown in Figure 4, panel B; the prevalence of target attainment is similar across measurements. The most common sequence patterns are shown in Figure 4, panel C. Sequence clustering analysis showed more women (OR 1.18, 95% CI 1.07-1.10) in the cluster with both the most measurements off target and the most LDL-c measurements furthest from the target.

The transition probabilities are shown in Table 3. Overall, patients had the highest probabilities (0.36-0.84) to remain in the initial LDL-c category, irrespective of the initial level of LDL-c.

Among these patients with related repeated measurements (N=10,771), 11,447 of 25,438 (45.00%) follow-up measurements remained on target or decreased to below the target threshold. We were able to assess factors associated with less favorable LDL-c change (ie, LDL-c that is stable but off target, decreased but not yet on target, or an increase in LDL-c) in a subset of 6871 measurements due to missing data on reported statin use (see Table 4). LDL-c values of women were more likely to increase or remain stable but off target (OR 1.44, 95% CI 1.30-1.59). Patients with diabetes more frequently succeeded in lowering LDL-c values below target or remaining on target (OR 0.72, 95% CI 0.63-0.82). Higher doses of statin, as well as higher doses in combination with a change in statin type, were associated with less favorable LDL-c change. The moment of prescription (ie, Was the statin change a response to the LDL-c measurement or a registration of pre-existing medication use?) could not be inferred from the data.

**Table 3:** Transition probabilities for low-density lipoprotein cholesterol (LDL-c) categories between measurement pairs.

LDL-c category at *first* measurement^a^	LDL-c category at next measurement, transition probability^b^					
	On target	<0.5 mmol/L	0.5-0.9 mmol/L	1.0-1.4 mmol/L	1.5-1.9 mmol/L	>2.0 mmol/L
On target	0.84	0.10	0.03	0.01	0.01	0.01
<0.5 mmol/L	0.30	0.52	0.12	0.04	0.02	0.01
0.5-0.9 mmol/L	0.23	0.19	0.43	0.09	0.03	0.02
1.0-1.4 mmol/L	0.20	0.12	0.16	0.39	0.08	0.05
1.5-1.9 mmol/L	0.19	0.13	0.11	0.12	0.36	0.10
>2.0 mmol/L	0.15	0.13	0.09	0.10	0.09	0.43

^a^The first measurement can be the first in a sequence as a whole or the first of a pair of measurements (eg, from the fourth to the fifth measurement).

^b^The transition probability is the probability a patient will be in one of the LDL-c categories at next measurement given the last measurement, which is the first of the pair.

**Table 4:** Logistic regression associations with deterioration of low-density lipoprotein cholesterol (LDL-c).

Characteristic			Odds ratio (95% CI)^a^
Age (per-year increase)			0.99 (0.99-1.00)
Women			1.44 (1.30-1.59)
Diabetes			0.72 (0.63-0.82)
Hypertension			0.93 (0.84-1.03)
Smoking (current)			1.00 (0.53-1.86)
**Statin change**			
	Same dose, same type	Reference	
	Same dose, different type	0.81 (0.58-1.13)	
	Higher dose, same type	1.82 (1.39-2.37)	
	Lower dose, same type	1.31 (0.93-1.85)	
	Higher dose, different type	1.47 (1.28-1.70)	
	Lower dose, different type	0.92 (0.80-1.06)	
Antithrombotic medication			0.81 (0.73-0.89)
Number of measurement			0.98 (0.93-1.03)
**Follow-up**			
	Short-term	Reference	
	Long-term	0.97 (0.88-1.08)	

^a^Total number of patients was 6871.

## Discussion

We evaluated the potential of routine clinical care data extracted from EHRs to obtain reliable information on LDL-c management in CVD patients referred to a tertiary care center. This approach may facilitate the implementation of a learning health care system, in which there is a constant cycle of data assembly, data analysis, interpretation, feedback, and change implementation. We showed that 51% of patients were not at their LDL-c target values at the time of referral. From a large proportion of patients, no follow-up LDL-c measurements (55%) were collected in our center. Patients with repeated measurements mostly showed no change in LDL-c level over time. The timing of drug prescription was difficult to determine from our data, limiting the interpretation of results regarding medication management.

Cardiovascular risk management, including LDL-c management, could substantially benefit from longitudinal evaluation of individual treatment trajectories. Cross-sectional studies, such as the EUROASPIRE IV, reported lower LDL-c target attainment compared to our findings [1], which may be explained by a difference in study population: the EUROASPIRE IV enrolled patients with coronary heart disease and patients at risk for CVD, which are defined as patients using blood pressure-, lipid-, or blood glucose-lowering medication. We also included patients with other CVD phenotypes in our main analyses, possibly increasing the prevalence of target attainment and thus explaining some of the differences with the EUROASPIRE IV. Also, we used the target in our national guideline (2.5 mmol/L), which is by definition less difficult to attain than 1.8 mmol/L. We found that patients with diabetes were less likely to be off target at baseline. In our center, we have a dedicated care program for diabetes run by diabetes nurses with structured, at least yearly, follow-up and clear protocol that includes LDL-c management.

Despite the compelling scientific evidence for the efficacy of LDL-c lowering in secondary prevention [7], LDL-c target attainment our secondary prevention cohort was poor. A review on CVRM guidelines found 21 guidelines with discrepancies in screening strategy and treatment target (1.8-2.5 mmol/L) [12]. Additional dedicated national guidelines exist—CVRM, chronic renal failure, and CVRM for the elderly—that all give different advice [10,13,14]. In some cases, multiple guidelines can apply, making it difficult for the clinician to decide which target value to strive for. Yet, despite the varying guidelines, our percentage attained targets remain low as compared to what guidelines dictate. The underlying mechanism remains to be solved, whether they be related to the physician, patient, process of clinical care and responsibilities, or insurance.

In our data, most patients remained in the same LDL-c category during every follow-up measurement. Possibly, attention for LDL-c management is limited in our tertiary care center, primarily focused on the complexity of disease and its comorbidity and, thus, LDL-c management might be more often delegated to the general practitioner. The large proportion of unique measurements (55%) and the finding that lower LDL-c target attainment was seen at baseline in patients with repeated measurements support this. Furthermore, treatment adherence due to polypharmacy—common in a tertiary population—might be challenging in our population [15]. In the Netherlands, health insurance is similar for all inhabitants, with clear equality, so differences between patient groups is unlikely to be attributed to differences in health care insurance. Based on our findings, the next step is in the implementation in clinical practice through, for example, a live dashboard, so that both patients and caregivers can view the findings and the comparisons between physicians. This may help to improve registration and patient care.

Our study has several strengths. We used routine clinical care data, including time and individual trajectories, for the evaluation of LDL-c management. We selected patients with manifest CVD without restrictions to phenotype—with a 100% accuracy of defining manifest CVD—treated in all departments within our center, making our results generalizable to a large population. We expected some confounding by indication, with patients with a higher LDL-c being more likely to be followed up in our center, which was confirmed by the difference at the first measurements. Yet, for our evaluation, this does not influence the validity but merely shows good clinical practice: complex patients with high LDL-c values are followed up in our specialist tertiary care center.

We also encountered some challenges. Our study population was based on LDL-c measurements and was selected based on diagnosis and intervention codes, which are incomplete due to registration issues as well as registration in different centers. This likely did not influence our results in terms of directions and magnitude of the outcome measures, yet decreased the sample size of the study population. Future analysis could possibly take the patient as a starting point, first selecting all patients with CVD and then extracting LDL-c data from these patients. This would enable the reporting, also, of the number of patients in whom LDL-c was not measured. Furthermore, 55% of our patients were only measured once; from our data, we cannot determine whether this was due to insufficient management or a change in clinician that was responsible for the CVRM. Information on discontinuation of care within our center is unavailable; this calls for combining different data sources, including general practitioner and pharmacy data [16]. This multidisciplinary care approach across health care providers is essential for the case of LDL-c and would potentially benefit from an LHS cycle that includes all caretakers involved in the care process. Lastly, medication registration was troublesome: no medication was registered among a large proportion of our patients and our data did not provide information on the timing of a prescription, only whether the prescription was registered at a certain date. Therefore, we could not determine whether medication at follow-up was newly prescribed as a response to the LDL-c measurement or whether it was merely registered. We cannot rule out that we might have classified patients as staying with the same statin and same dosage who, in fact, received the medication just after the first consult. This would explain why increase in statin dose was associated with a less favorable change in LDL-c; it might have actually been the right clinical response to an insufficient LDL-c level. Thus, the effect of statin change may have been underestimated.

The EHR is a system primarily designed for registration of care. In clinical notes, clinicians register the clinical pathway of patients, including symptoms, measurements, and considerations of treatments. These considerations, in particular (ie, interpretation of data that leads to decisions), are difficult to capture within data extractions from the EHR. Harmonized clinical pathways with special attention to structured data collection are key for the availability and extractability of reliable data. Therefore, The Center for Circulatory Health of the UMC Utrecht initiated the Utrecht Cardiovascular Cohort (UCC) [17]. Traditional cardiovascular risk factors, according to the Dutch CVRM guidelines, are collected for all patients at all departments treating CVD patients and are registered in a structured form within the EHR [10,17]. To further develop the LHS, we need to design and implement feedback routes to feed back the evidence we generate. Computerized decision support systems (CDSSs) that help guide CVRM are increasingly developed to facilitate live data analysis, interpretation, and guideline-adherent therapy advice [18-20]. These CDSSs seem promising in improving cardiovascular risk factors, especially when embedded in the EHR [21,22]. Also, structured registration of CVRM and outcomes would enable the estimation of cost-effectiveness, which, up to now, is mostly based on simulation studies; eventually, this will provide better, value-based health care [23].

In conclusion, routine clinical care data can be used to obtain insights into clinical questions such as LDL-c target attainment and can be tailored into feedback from individual patients and clinicians. Our routine clinical care data, with high off-target prevalence, insufficient uptake of the guideline change, and little change in LDL-c over time, showed that improvement in guideline adherence is needed. Registrations of diagnosis, follow-up trajectory, and medication use need to be improved in order to enhance the usability of the EHR system for these types of questions.

## Appendix

Multimedia Appendix 1 Supplementary tables: Tables MA1-1 and MA1-2.

## Abbreviations

CDSS: computerized decision support system
CKD: chronic kidney disease
CVD: cardiovascular disease
CVRM: cardiovascular risk management
EHR: electronic health record
EUROASPIRE: European Action on Secondary and Primary Prevention by Intervention to Reduce Events
LDL-c: low-density lipoprotein cholesterol
LHS: learning health care system
OR: odds ratio
SURF: SUrvey of Risk Factor management
UCC: Utrecht Cardiovascular Cohort
UMC: University Medical Center
UPOD: Utrecht Patient-Oriented Database
