# Supplementary material for: Low-Density Lipoprotein Cholesterol Target Attainment in Patients With Established Cardiovascular Disease: Analysis of Routine Care Data
Source: JMIR Med Inform. 2020 Apr 2;8(4):e16400. doi: 10.2196/16400 (PMC7163416; doi:10.2196/16400)

**Multimedia Appendix 1**

**Table MA1-1. Reference table atorvastatin equivalent dose**

According to the Dutch guideline of cardiovascular risk management, first choice cholesterol lowering drug is simvastatin 40mg. This corresponds to atorvastatin 20mg. Therefore all medications are transformed into atorvastatin 20mg equivalent dose.

| Statin type | Atorvastatin 20mg equivalent |
| --- | --- |
| Simvastatin | 40 mg |
| Lovastatin | 80 mg |
| Pravastatin | 80 mg |
| Fluvastatin | 160 mg |
| Rosuvastatin | 10 mg |

<https://www2.gov.bc.ca/assets/gov/health/health-drug-coverage/pharmacare/rdp_decisiontree_statins.pdf>

**Table MA1-2. Median LDL-c values of baseline measurements per year**


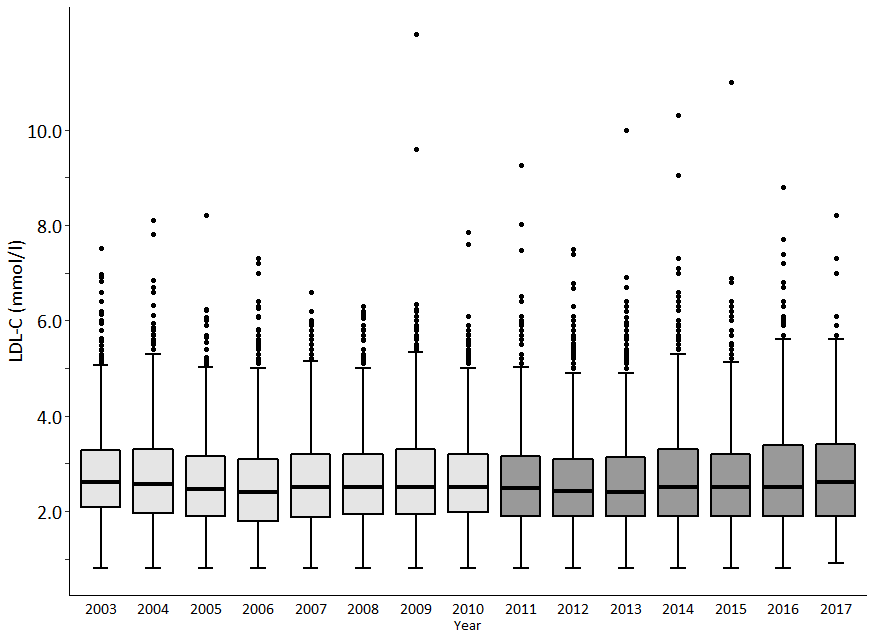

Supplement: Multimedia Appendix 1 [file medinform_v8i4e16400_app1.docx]
